# Integrative Bioinformatics Analysis Reveals That Infarct-Mediated Overexpression of Potential miR-662/CREB1 Pathway-Induced Neuropeptide VIP Is Associated with the Risk of Atrial Fibrillation: A Correlation Analysis between Myocardial Electrophysiology and Neuroendocrine

**DOI:** 10.1155/2021/8116633

**Published:** 2021-11-22

**Authors:** Pengpai Zhang, Bo Liu

**Affiliations:** Department of Cardiology, Xinhua Hospital, School of Medicine, Shanghai Jiaotong University, Shanghai, China

## Abstract

**Background:**

Neuropeptide levels are closely associated with the development and maintenance of atrial fibrillation (AF) after myocardial infarction (MI). This study was aimed at investigating the regulatory network that affects neuropeptide expression through transcription factor modulation.

**Methods:**

We downloaded three datasets from the GEO database, and after performing differential and crosstabulation analyses, we screened out differentially expressed (DE) miRNAs and DEmRNAs coexpressed in AF and MI and performed DEmiRNA–DEmRNA pairing prediction; from which, we constructed a regulatory network. Subsequently, the hsa-miR-662-CREB1-VIP axis was obtained, and the role of CREB1 and VIP in the development of AF after MI was further revealed by single-cell analysis and prediction model construction.

**Results:**

In this study, eight DEmRNAs and four miRNAs were screened. hsa-miR-662 was identified by database integration analysis to regulate the transcription factor CREB1, a potential transcriptional regulator in VIP. CREB1 and VIP are mainly enriched in pathways of energy metabolism, ion channels, and myocardial contraction. CREB1 and VIP were identified as biomarkers of the onset and prognosis of MI and AF.

**Conclusions:**

In this study, the miR-662/CREB1/VIP regulatory pathway was constructed through integrated analysis of datasets, thus providing new ideas to study the mechanisms of AF development.

## 1. Introduction

Ischaemic heart disease (IHD) is a major threat to human health, and the rate of disability and mortality owing to IHD has been exponentially increasing worldwide since 1990, with approximately 82% of deaths and 89% of disability occurring because of IHD in developing countries [1]. Acute myocardial infarction (AMI) is one of the deadliest cardiovascular diseases, and it is also the leading cause of death in people over 40 years of age in Europe and the United States. AMI constitutes 33% of deaths due to coronary heart disease [2].

AMI is often concomitant with various types of arrhythmias; among which atrial fibrillation (AF) is relatively common, and the incidence of AF after AMI is 6% to 20% [3]. The incidence of in-hospital AF is 6% to 8.4% [4]. AF significantly reduces the quality of life of patients and increases the risk of stroke by 1.6–3.5 times, thus increasing the death rate by 50% to 90% [5, 6]. In recent years, researchers have devoted themselves to exploring the mechanisms of AF at the cellular and molecular levels to identify more accurate therapeutic targets. Atrial fibrosis is an important pathophysiological factor in the development of AF and is closely associated with recurrence and complications of AF. Atrial fibrosis is the deposition of abnormal collagen fibres in the interstitial matrix of the atrial cells. The normal extracellular matrix (ECM) mainly consists of type I collagen and a small amount of type II collagen; type III collagen levels increase in AF. Atrial fibrosis causes the onset of reentrant circuits through inhomogeneous conduction, leading to AF [7]. Multiple signalling pathways and alterations in multiple factors are collectively involved in the process of atrial fibrosis [8, 9].

Recent studies have found that the expression of various neuropeptides is altered during the onset of AF. For example, in patients with myocardial infarction and animal models of cardiac ischaemia, plasma concentrations of CGRP were significantly increased [10–13]. CGRP and intermedin (IMD) modulate cardiac performance during recovery from acute myocardial ischaemia and reperfusion [14, 15]. The expression of *CRLR* and *RAMP3* genes increases in both the early and late phases of reperfusion, and the number of IMD binding sites in the myocardium increases during ischaemia [13, 15]. These findings suggest that IMD protects the heart in response to ischaemic injury. Neuropeptide Y (NPY) is a sympathetic neurotransmitter that coexists with catecholamines in sympathetic fibres and has been found to play an important role in the changes in arrhythmia pathology. Alterations in its levels and activity are associated with the development of various cardiovascular diseases such as cardiac hypertrophy, arrhythmias, heart failure, atherosclerosis, ischaemia, and hypertension [16]. However, the neuropeptides associated with the development of AF after MI have not yet been reported in detail. The regulation of the expression of the relevant neuropeptides and the mechanisms by which they function remain to be elucidated.

MicroRNAs (miRNAs) play an important role in myocardial remodelling, myocardial cell necrosis, and reperfusion. Several miRNAs target the membrane receptor system and are directly involved in AF as well as cardiac arrhythmias [17–19]. Previous studies have demonstrated that the expression of neuropeptides is regulated by miRNAs [20]. Moreover, numerous circulating miRNAs have been reported as potential biomarkers for reactive oxygen species- (ROS-) related heart disease, such as miRNA-499, miRNA-199, miRNA-21, miRNA-144, miRNA-208a, and miRNA-34a [21]. We, therefore, hypothesised that miRNAs play a role in the development of AF after MI by regulating the expression of neuropeptides.

miRNAs and transcription factors (TFs) are two key gene regulators involved in many important cellular processes, including cell differentiation, proliferation, and apoptosis. miRNAs primarily regulate gene expression at the posttranscriptional level, whereas TFs regulate gene transcription at the transcriptional level. Researchers have demonstrated that miRNAs and TFs can synergistically regulate the same target genes and may regulate each other [22]. Changes in the levels of neuropeptides are also regulated by TFs [23, 24]. We can, therefore, speculate that miRNAs may influence neuropeptide expression by regulating TFs.

Neuropeptide levels are closely related to the occurrence and maintenance of AF after MI. This study was aimed at investigating the regulatory network of miRNAs that may influence the expression of neuropeptides through the regulation of TFs to reveal the molecular biological mechanism of arrhythmia development after MI, identifying targets for arrhythmia diagnosis and treatment, and providing new ideas for clinical diagnosis and treatment.

## 2. Materials and Methods

### 2.1. Download and Preprocessing of Datasets from the GEO Database

For this study, we downloaded three datasets from the Gene Expression Omnibus (GEO) database (https://www.ncbi.nlm.nih.gov/geo/). The mRNA and miRNA microarray data of the right auricle from patients with AF were downloaded from GSE2240 and GSE68475, respectively [25, 26]. We used the “limma” package to identify differentially expressed genes (DEGs) between the two groups. By analysing the GSE76591 dataset, miRNAs that were differentially expressed between the MI and normal groups were identified [27]. The GSE2240 dataset contained 30 right atrium tissue samples, of which 10 were from patients with AF. The GSE68475 dataset contained 20 right atrium tissue samples, of which 11 were from patients with AF. The GSE76591 dataset contained 21 right atrium tissue samples, of which 9 were from patients with AF and 12 were from normal tissues (control group). Gene expression data were merged, and gene identification numbers were transformed using Perl (https://www.perl.org/) to obtain a gene expression matrix. Depending on the platform and data type of the microarray, the data were considered for log2 conversion. Subsequently, the “normalizeBetweenArrays” function of the “limma” package was applied to normalise the samples within the chip, and the “boxplot” function was used to verify the removal of intersample differences. Lastly, we used a preprocessed gene expression matrix for neuropeptide gene pooling.

We wanted to select a broad list of candidates for neuropeptide genes. Therefore, we browsed through Google and Wikipedia (https://en.wikipedia.org/wiki/Neuropeptide) and reviewed the literature. Neuropeptide Y (NPY) [28], substance P (SP), CGRP [29], atrial natriuretic peptide (ANP), brain natriuretic peptide (BNP), C-type natriuretic peptide (CNP) [30], pituitary adenylate cyclase-activating polypeptide (PACAP) [31], secretoneurin (SN) [32], galanin [33], vasoactive intestinal polypeptide (VIP) [34], and copeptin [35] were included in this study. Eventually, we selected a cardiac-associated neuropeptide gene set that contained neuropeptides and their receptors.

### 2.2. Gap Analysis

Based on the grouping information of the clinical samples, we used the “limma” package to identify differentially expressed genes (DEGs) between the two groups. Upregulated and downregulated genes were identified based on the fold change scores. The upregulated and downregulated DEGs obtained from differential analysis are displayed in the volcano plot. Furthermore, differentially expressed (DE) neuropeptide mRNAs were screened and labelled in the volcano map. We used intersection analysis to screen out DEmiRNAs that were coexpressed in AF and MI for subsequent ceRNA network analysis. The “VennDiagram” package of R software (https://cran.r-project.org/web/packages/VennDiagram/index.html) was used to plot a Venn diagram to demonstrate the results of intersection analysis.

### 2.3. Regulatory Network Construction and Target Gene Establishment

Based on the information of TF–mRNA matching data in the TTRUST database, the DETF–DEmRNA relationship pairs were obtained by pairing the DEmRNAs in AF with the corresponding DETFs. Furthermore, the DETF–DE neuropeptide mRNA relationship pairs were identified. We then performed DEmiRNA–DEmRNA pairing predictions for coexpressed DEmiRNAs and DEmRNAs in AF and MI according to the miRWalk website (http://mirwalk.umm.uni-heidelberg.de/). Lastly, using the Cytoscape (version 3.8.2) software, DEmiRNA–DETF, DEmiRNA–DEmRNA, and DETF–DEmRNA pairs were selected to construct regulatory networks. The miRNA–TF–mRNA axis obtained by network visualisation analysis was selected for subsequent analysis. Based on the DGIdb database (https://dgidb.org/), TFs and neuropeptides in the selected miRNA–TF–mRNA axis were used as targets to be matched with drugs to screen for potential targeting drugs. The protein-protein interaction (PPI) network was constructed by the STRING web tool [36]. The potential relationships of interaction between drugs and target genes were visualised on Sankey plots. Furthermore, using the Find Individual Motif Occurrences (FIMO) web and downloaded motif files, TF motifs were predicted [37].

### 2.4. Enrichment Analysis of Pathways

To understand the functional pathways that are altered during AF and MI, the Kyoto Encyclopedia of Genes and Genomes (KEGG) and gene ontology (GO) analyses were performed using the “http://org.Hs.eg.db” and “clusterProfiler” packages to identify potential upregulated or downregulated functional pathways [37]. The GO terms were divided into three main categories, namely, biological process (BP), cellular component (CC), and molecular function (MF). We used Gene Set Enrichment Analysis (GSEA) to determine the active pathways that differed between the high- and low-expression groups of the target genes. We first estimated the difference in mean mRNA levels per gene between the two groups. Subsequently, fold change scores were calculated to rank the genes. The KEGG and GO datasets were used for GSEA separately. We selected false discovery rate (FDR) and adjusted *P* value as a filter for differentially activated pathways. Lastly, bubble plots or bar charts were used to present the results of these enrichment analyses.

### 2.5. Single-Cell Analysis

Single-cell RNA-seq data of arterial and cardiac tissues were downloaded for analysing target gene expression at the single-cell level [38, 39]. First, single-cell RNA-seq data were qualitatively analysed to remove ineligible cells and genes. Genes expressed in at least three cells were defined as eligible [40]. Mitochondrial genes were removed. In previous studies, the “Seurat” package was used to perform tSNE downscaling of the filtered RNA-seq and the “SingleR” package was used for cell cluster annotation [41, 42]. Lastly, the expression of the target genes at the single-cell level was annotated on the tSNE plot in the form of a heat map.

### 2.6. Clinical Predictive Modelling Analysis

To determine the predictive and representative ability of the selected genes for different cardiac states, we first analysed their expression in the AF and sinus rhythm (SR) groups. The Wilcoxon rank-sum test was used for statistical analysis of differences in gene expression between the two groups. Furthermore, the Spearman correlation analysis was used to analyse the correlation between the selected genes. Lastly, all selected genes were separately incorporated into logistic regression analyses to construct AF prediction models as previous research [43]. Receiver operating characteristic (ROC) curves were used to check the reliability of each model. The area under curve (AUC) values were in the range of 0.5–1, with values closer to 1 indicating better prediction. When AUC was between 0.7 and 0.9, the model exhibited a reliable predictive power. All statistical analyses were plotted using the R software (version 4.0.5).

## 3. Results

### 3.1. Differentially Expressed mRNAs in AF

**Figure 1:**
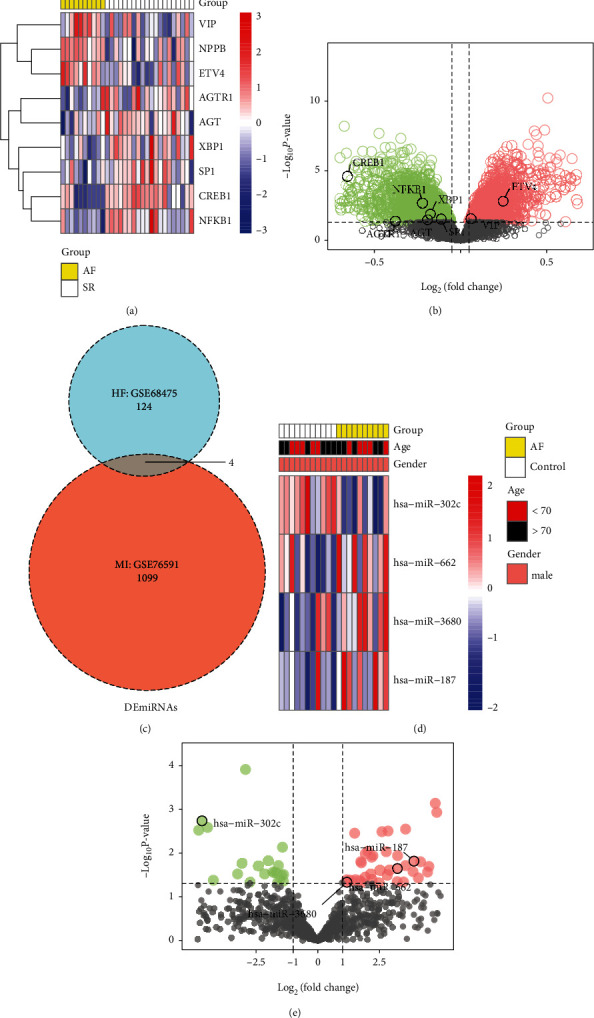
Identification of DE neuropeptide mRNAs and miRNAs. (a) Heat map of DE neuropeptide mRNAs in the AF and SR groups. (b) Volcano plot showing FC scores and *P* values of DEGs in the AF and SR groups. DE neuropeptide mRNAs are also labelled in the plot. (c) Venn diagram showing the number of DEmiRNAs shared in the MI control and AF groups. (d) Heat map showing the expression of shared DEmiRNAs in AF samples. (e) Volcano plot showing the results of analysis of DEmiRNAs in the AF group. Shared DEmiRNAs between MI and AF are labelled in the plot.

The flow chart for the analysis of the study is demonstrated in Supplementary Figure 1. Analysis of variance revealed 3709 DEmRNAs between the AF and control groups. A total of 2041 upregulated and 1668 downregulated genes were identified based on fold change scores. Of these, there were eight DE neuropeptides or their receptors, namely, NPR3, NPPB, NPR1, CALCR, NPR2, VIP, AGT, and AGTR1. Of the DEmRNAs found in AF, five TFs (ETV4, XBP1, SP1, CREB1, and NFKB1) and four regulated neuropeptides (VIP, NPPB, AGTR1, and AGT) or their receptors were identified and constituted seven TF-mRNA pairs. The heat map of these eight DEmRNAs demonstrated that their expression patterns were consistent between the AF and control groups (Figure 1(a)).  Figure 1(b) demonstrates the results of DEmRNA differential analysis, and the eight neuropeptide-related DEmRNAs are labelled in the volcano map.

### 3.2. Differentially Expressed miRNAs in MI and AF

**Figure 2:**
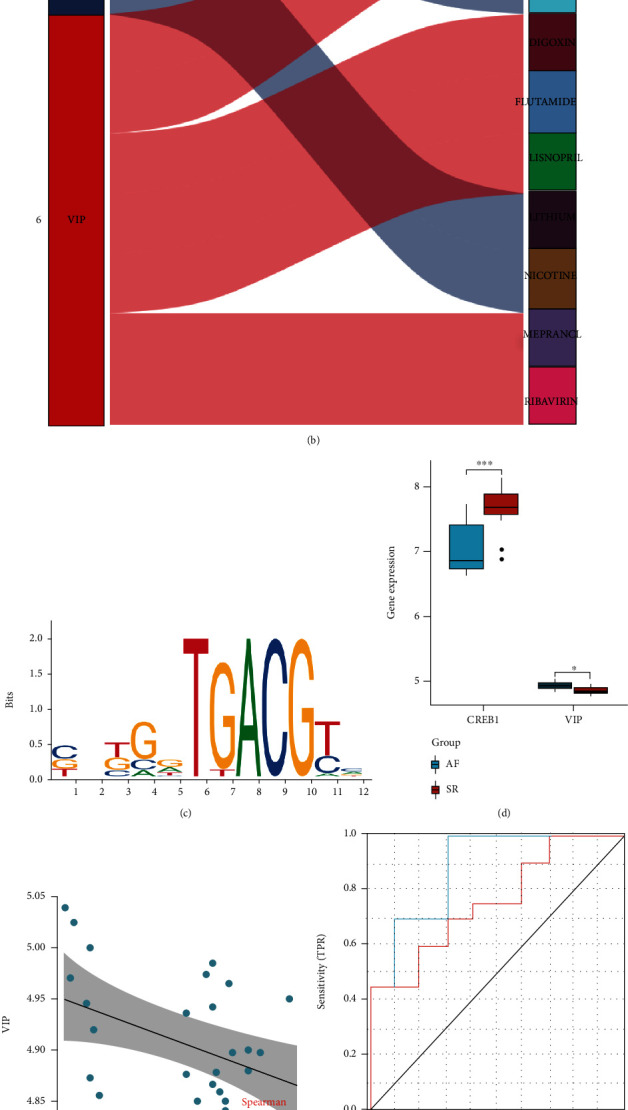
Protein interaction networks and drug prediction analysis. (a) Network showing the regulatory relationship between DEmiRNAs and DEmRNAs in AF. Red boxes show the constructed miRNA–TF–mRNA regulatory axis for CREB1 and VIP. (b) Sankey diagram showing predicted drugs that target CREB1 and VIP. (c) The motif of CREB1. (d) Differential expression of CREB1 and VIP in AF and SR. (e) Correlation analysis of VIP and CREB1. (f) Probability of CREB1 and VIP to predict the occurrence of AF. (g) Reliability of the joint CREB1 and VIP prediction model.

Based on differential expression analysis of genes, 124 DEmiRNAs and 1099 DEmiRNAs were obtained from AF and MI data, respectively (Figure 1(c)). Based on the coanalysis of DEmiRNAs in AF and MI, four shared DEmiRNAs were identified, namely, hsa-miR-302c, hsa-miR-662, hsa-miR-3680, and hsa-miR-187. The heat map demonstrated the relative expression of these four DEmiRNAs in the AF samples (Figure 2(d)).  Figure 1(e) demonstrates the distribution of *P* values and log2FC corresponding to the genes expressed in AF. Of these genes, the expression of hsa-miR-302c was downregulated, whereas that of hsa-miR-662, hsa-miR-3680, and hsa-miR-187 expression was upregulated.

### 3.3. KEGG and GO Functional Enrichment Analysis of DEmRNA

**Figure 3:**
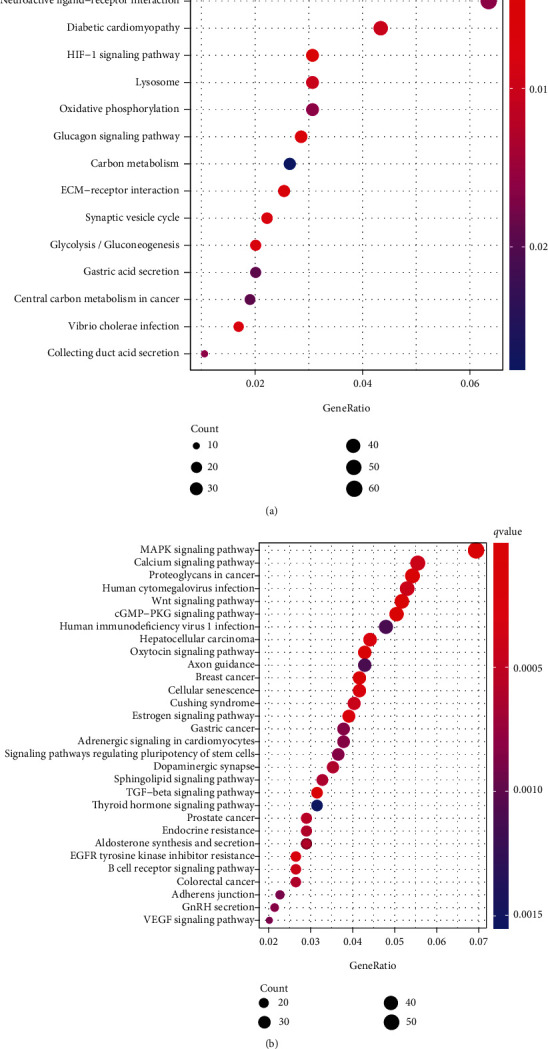
KEGG pathway analysis of DEmRNAs in the AF group: (a) upregulated KEGG pathways in the AF group; (b) downregulated KEGG pathways in the AF group.

All upregulated and downregulated DEmRNAs were included in the GO and KEGG functional enrichment analyses. Enrichment analysis of 2041 upregulated DEmRNAs revealed 14 KEGG pathways that were found to be enriched in AF. Of the 14 pathways, those associated with myocardial activity and neuropeptide regulation included hypoxia-inducible factor-1- (HIF-) 1 signalling pathway, ECM-receptor interaction, glucagon signalling pathway, diabetic cardiomyopathy, neuroactive ligand–receptor interaction, and oxidative phosphorylation. These 14 pathways were revised upregulated in AF (Figure 3(a)). The energy metabolism-related pathways suggest that electrophysiological disturbances in AF are caused by disturbances in energy metabolism. In addition, 96 KEGG pathways were found to be downregulated in AF based on enrichment analysis of the functions of 1668 downregulated genes. Wnt signalling pathway, calcium signalling pathway, adherens junctions, vascular smooth muscle contraction, and other downregulated pathways were associated with decreased myocardial contraction and electrophysiological disturbance activity (Figure 3(b)). In addition, similar functional pathways were identified by GO analysis (see Supplementary Figure 2).

### 3.4. Network Construction

These DEmiRNA–DEmRNA, DETF–DEmRNA, and DEmiRNA–DETF pairs were imported to the Cytoscape software to construct a DEmiRNA–DETF–DEmRNA interoperability network (Figure 2(a)). The results revealed that hsa-miR-662 regulated the TF CREB1. PPI network of SP1, AGT, NPPB, NFKB1, CREB1, XBP1, AGTR1, ETV4, and VIP is shown in supplementary figure 3. Significant associations were found between CREB1, NFKB1, and SP1 in the two subnetworks. Based on the visual analysis of this network, the hsa-miR-662-CREB1-VIP axis was the only regulatory axis that contained miRNA, TF, and mRNA. The Sankey diagram demonstrated CREB1 and VIP with their respective targeted drugs (Figure 2(b)). According to the predicted results, CREB1 interacts with citalopram, lithium, nicotine, and alcohol, and VIP interacts with ribavirin, azaserine, lisinopril, androstanolone, omeprazole, flutamide, and digoxin. The motif sequence of CREB1 is shown in Figure 2(c).

### 3.5. Expression of CREB1 and VIP in Tissues and Predictive Model Construction

Statistically significant differences were found in the expression levels of CREB1 and VIP between the AF and SR groups (Figure 2(d)). The expression of CREB1 was higher in the SR group than that in the AF group, whereas the expression of VIP was lower in the SR group than that in the AF group. A negative correlation was observed between CREB1 and VIP expression in AF (Figure 2(e)). The prediction models constructed based on logistic regression demonstrated that CREB1 (AUC = 0.885) and VIP (AUC = 0.76) both exhibited adequate accuracy in predicting AF (Figure 2(f)). The inclusion of CREB1 and VIP in the prediction model also exhibited adequate predictive accuracy (AUC = 0.88, confidence interval (CI) = 0.741–1; Figure 2(g)). The smaller the prediction score, the higher the likelihood of obtaining an AF sample.

### 3.6. Single-Cell Analysis

**Figure 4:**
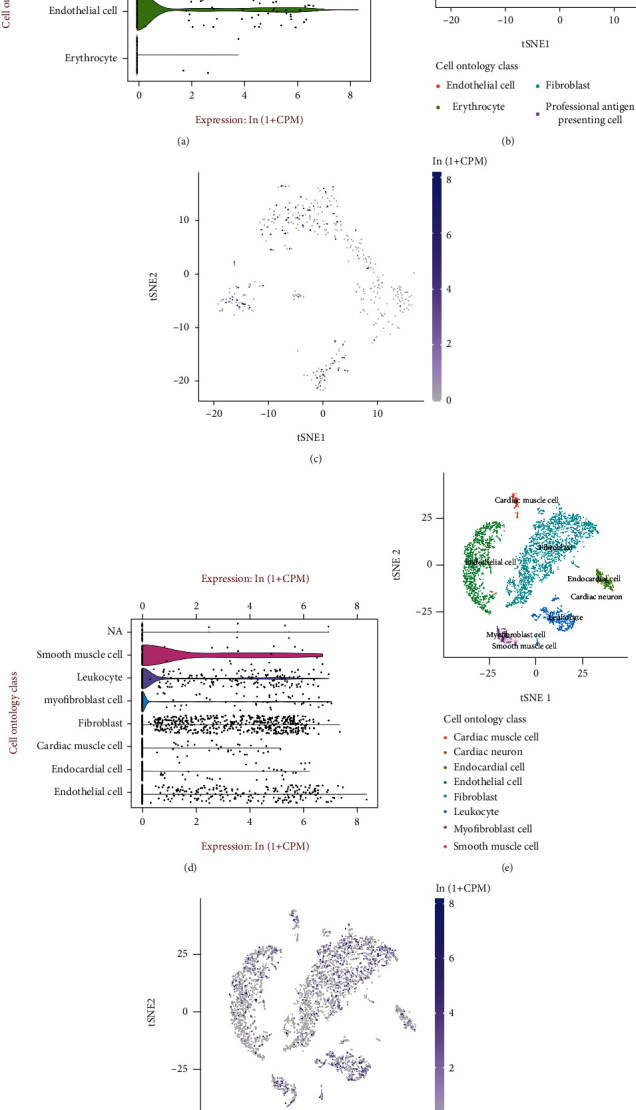
Single-cell analysis of arterial and cardiac tissues: (a) quality control results of the analysis of single-cell RNA-seq in arterial tissues; (b) a tSNE plot of single-cell descending in arterial tissues; (c) CREB1 expression at the single-cell level in arterial tissues; (d) quality control results of the analysis of single-cell RNA-seq in cardiac tissues; (e) single-cell descending tSNE maps in arterial tissues; (f) CREB1 expression in single-cell clusters in cardiac tissues.

First, the filtered single-cell RNA-seq data were qualitatively tested; the results are demonstrated in Figures 4(a) and 4(d). The tSNE plots of single-cell RNA-seq descending for arterial and cardiac tissues and the cell cluster annotations are demonstrated in Figures 4(b) and 4(e), respectively.  Figure 4(c) demonstrates that CREB1 is highly expressed in some endothelial cells, fibroblasts, and professional antigen-presenting cells in arterial tissues.  Figure 4(f) demonstrates that CREB1 is highly expressed in some cardiac muscle cells, cardiac neurons, endocardial cells, and endothelial cells in cardiac tissues. The results suggest that CREB1 plays a complex role in all types of heart cells.

### 3.7. Results of the GSEA for CREB1 and VIP

**Figure 5:**
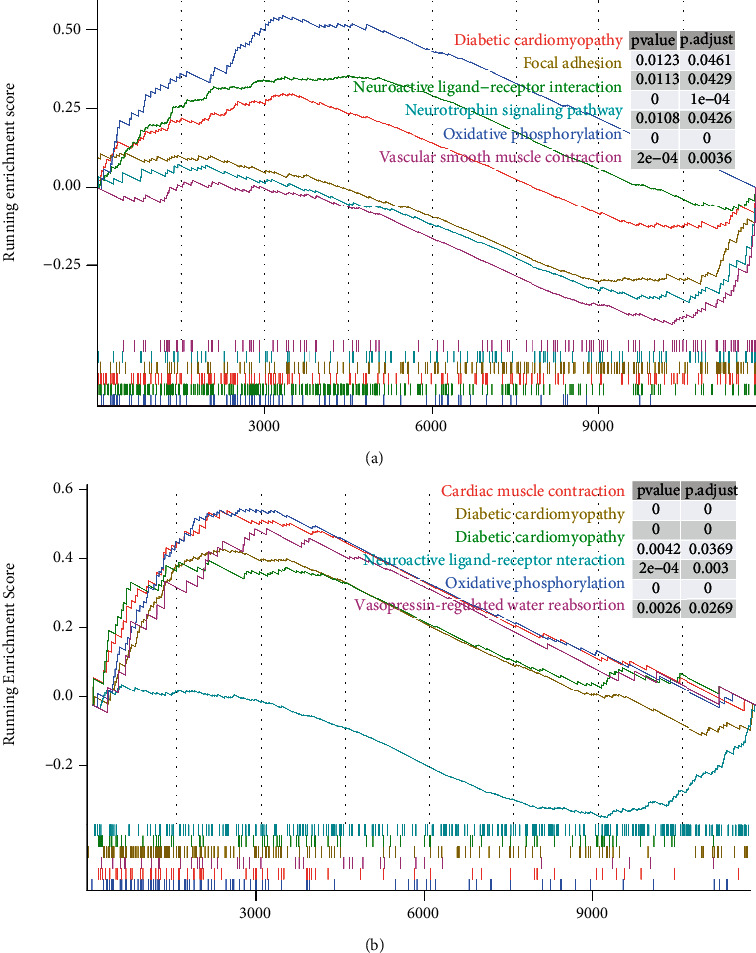
GSEA of CREB1 and VIP in AF: (a) pathways in AF in which the CREB1 high-expression group is enriched in the KEGG gene set; (b) pathways in AF in which the VIP high-expression group is enriched in the KEGG gene set. Curve peaks above the horizontal axis indicate that the pathway is upregulated in AF; otherwise, it represents downregulation.

GSEA results demonstrated that the KEGG pathways associated with CREB1 were those involved in oxidative phosphorylation, neuroactive ligand–receptor interaction, and vascular smooth muscle contraction (Figure 5(a)), and the GO pathways associated with CREB1 and VIP were those involved in inhibitory extracellular ligand-gated ion channel activity, ligand-gated anion channel activity, and ATP synthesis coupled electron transport (Supplementary Figure 4A). The results suggest that CREB1 is associated with electrophysiological activities of cardiomyocytes, such as ion channel activity, energy metabolism, and vascular smooth muscle contraction. GSEA revealed that the set of KEGG genes mainly associated with VIP in AF was those involved in oxidative phosphorylation, cardiac muscle contraction, and neuroactive ligand–receptor interaction (Figure 5(b)); and the GO genes that were primarily associated with VIP were those involved in the regulation of oxidative stress-induced neuron death, cardiac myofibril assembly, and cardiac muscle cell action potential (Supplementary Figure 4B). In conclusion, CREB1 and VIP share abnormal pathways related to energy metabolism, ion channels, and cardiovascular smooth muscle contraction.

## 4. Discussion

This study was aimed at constructing a potential miRNA–TF–neuropeptide regulatory network, thereby revealing the molecular biological mechanisms underlying the development of AF after MI, and at identifying new biological targets for the diagnosis and treatment of AF for improving prognosis.

To construct a potential miRNA–TF–neuropeptide regulatory network, we performed a differential analysis. Between the AF and control groups, we found eight DEmRNAs for neuropeptides or their receptors, which were NPR3, NPPB, NPR1, CALCR, NPR2, VIP, AGT, and AGTR1. Analysis of the GSE68475 and GSE76591 datasets revealed the following four DEmiRNAs in the AF and MI groups: hsa-miR-302c, hsa-miR-662, hsa-miR-3680, and hsa-miR-187. Subsequently, based on database integration analysis, hsa-miR-662 was found to regulate CREB1, which is a potential transcriptional regulator of VIP. Therefore, a new miR–662/CREB1/VIP pathway was proposed. Furthermore, using the DGIdb database, potential target drugs for CREB1 and VIP were also predicted. CREB1 may be a potential target of citalopram, lithium, nicotine, and alcohol. VIP may be a potential target of ribavirin, azaserine, lisinopril, androstanolone, omeprazole, flutamide, and digoxin. GSEA revealed that, of the pathways in which CREB1 and VIP are jointly involved, abnormalities occurred mainly in pathways related to energy metabolism, ion channels, and myocardial contraction. Further analysis suggested that CREB1 and VIP were significantly more highly expressed in both MI and AF groups. ROC curve analysis suggested that CREB1 and VIP can be used as biomarkers for the occurrence and prognosis of AF and MI.

VIP is a neuropeptide that belongs to the glucagon/secretin superfamily and is a ligand for class II G protein-coupled receptors [44, 45]. VIP acts extensively, stimulating cardiac contraction, causing vasodilatation, promoting neuroendocrine-immune communication, increasing glycogenolysis, and decreasing arterial blood pressure [44–47]. The release of VIP from neurons can lead to changes in the electrophysiological properties of the atria, which induce and maintain AF [34, 48]. Furthermore, during the acute phase of infarction, there is a significant increase in the release of VIP from the sympathetic nerves of the heart [49]. In line with the results of a previous study, the current study found that VIP played an important role in the occurrence of AF and MI. The current study identified potential relationships between VIP and ribavirin, azaserine, lisinopril, androstanolone, omeprazole, flutamide, and digoxin, suggesting that these drugs have the potential to target VIP for the treatment of AF and MI.

CREB1 is an abundantly expressed TF that has been reported to be involved in the regulation of various physiological functions. CREB1 affects target genes by binding to specific cAMP response elements in the promoter region of the target gene, thereby activating gene transcription. CREB1 proteins may also directly interact with target proteins. CREB1 has been demonstrated to play a key role in the maintenance of cardiac rhythm and cardiac contraction [50]. CREB1 is downregulated in the infarcted region when MI occurs. If CREB1 is overexpressed after the onset of MI, cardiac dysfunction after AMI can be alleviated [51]. The results suggested a potential relationship between CREB1 and citalopram, lithium, nicotine, and alcohol. Identifying the signalling pathways that regulate gene expression in heart diseases may help in identifying new molecular targets for the development of effective drugs.

miRNAs are protected from degradation when released into circulation; therefore, they are considered eligible biomarkers. Recent studies have demonstrated the potential of miRNAs as biomarkers in various cardiovascular disorders [52, 53]. As regulators of cardiac cell growth and differentiation, they are likely to play a role in the pathogenesis of cardiovascular diseases. In the present study, hsa-miR-662 was found to have a regulatory role on CREB1.

Furthermore, significant associations were found between CREB1, NFKB1, and SP1 in the two subnetworks. In previous studies, NFKB1 and SP1 were also found to be associated with myocardial pathological hypertrophy and regulated by miRNAs [54]. SP1 and NFKB1 were also identified in a previous study as key transcription factors that are regulated after myocardial exposure to mechanical stress [55]. Exercise is an important way to promote human health [56, 57]. Exercise training was also found to improve the expression of SP1 and NFKB1 in the heart [58, 59].

In this study, integrative bioinformatics analysis revealed that infarct-mediated overexpression of potential miR-662/CREB1 pathway-induced neuropeptide VIP may be associated with the risk of atrial fibrillation. The association between myocardial electrophysiology and neuroendocrine was also revealed in the present study. CREB1 activity may not be determined only by expression of CREB1. Therefore, further experimental studies are required to validate and understand the specific mechanism of action of CREB1 and VIP.

## 5. Conclusions

In this study, the miR-662/CREB1/VIP regulatory pathway was successfully constructed by integrating AF and heart attack datasets. CREB1 and VIP, as prognostic biomarkers for the development of AF and MI, were found to be enriched mainly in the pathways related to energy metabolism, ion channels, and myocardial contraction, thus providing a new avenue for investigating the mechanisms of AF development.

## Data Availability

A total of three datasets (including GSE2240, GSE68475, and GSE76591) were downloaded from the Gene Expression Omnibus database (https://www.ncbi.nlm.nih.gov/geo/) and applied to this study.

## Abbreviations

AF: Atrial fibrillation
SR: Sinus rhythm
MI: Myocardial infarction
VIP: Vasoactive intestinal peptide
CREB1: cAMP-response element binding protein.

## Supplementary Materials

Supplementary Materials All supplementary diagrams are included in the “Supplementary figures” file.
