# Supplementary material for: Integrative Bioinformatics Analysis Reveals That Infarct-Mediated Overexpression of Potential miR-662/CREB1 Pathway-Induced Neuropeptide VIP Is Associated with the Risk of Atrial Fibrillation: A Correlation Analysis between Myocardial Electrophysiology and Neuroendocrine
Source: Dis Markers. 2021 Nov 22;2021:8116633. doi: 10.1155/2021/8116633 (PMC8629660; doi:10.1155/2021/8116633)
Supplement: Supplementary Materials — All supplementary diagrams are included in the “Supplementary figures” file. [file 8116633.f1.doc]

# Supplementary Figure Legends


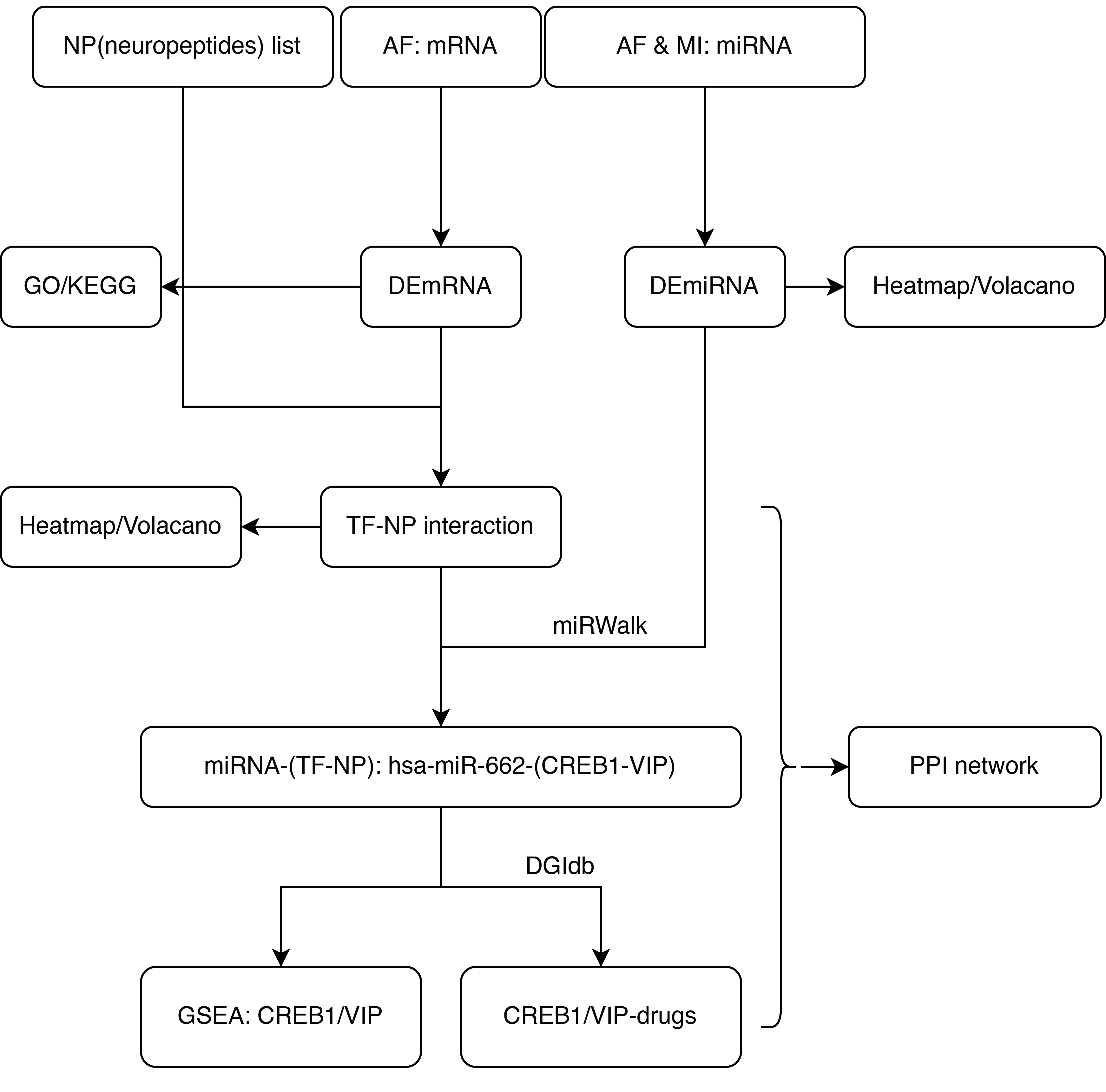


Supplementary Figure 1. Flow chart of the analysis process for this study.


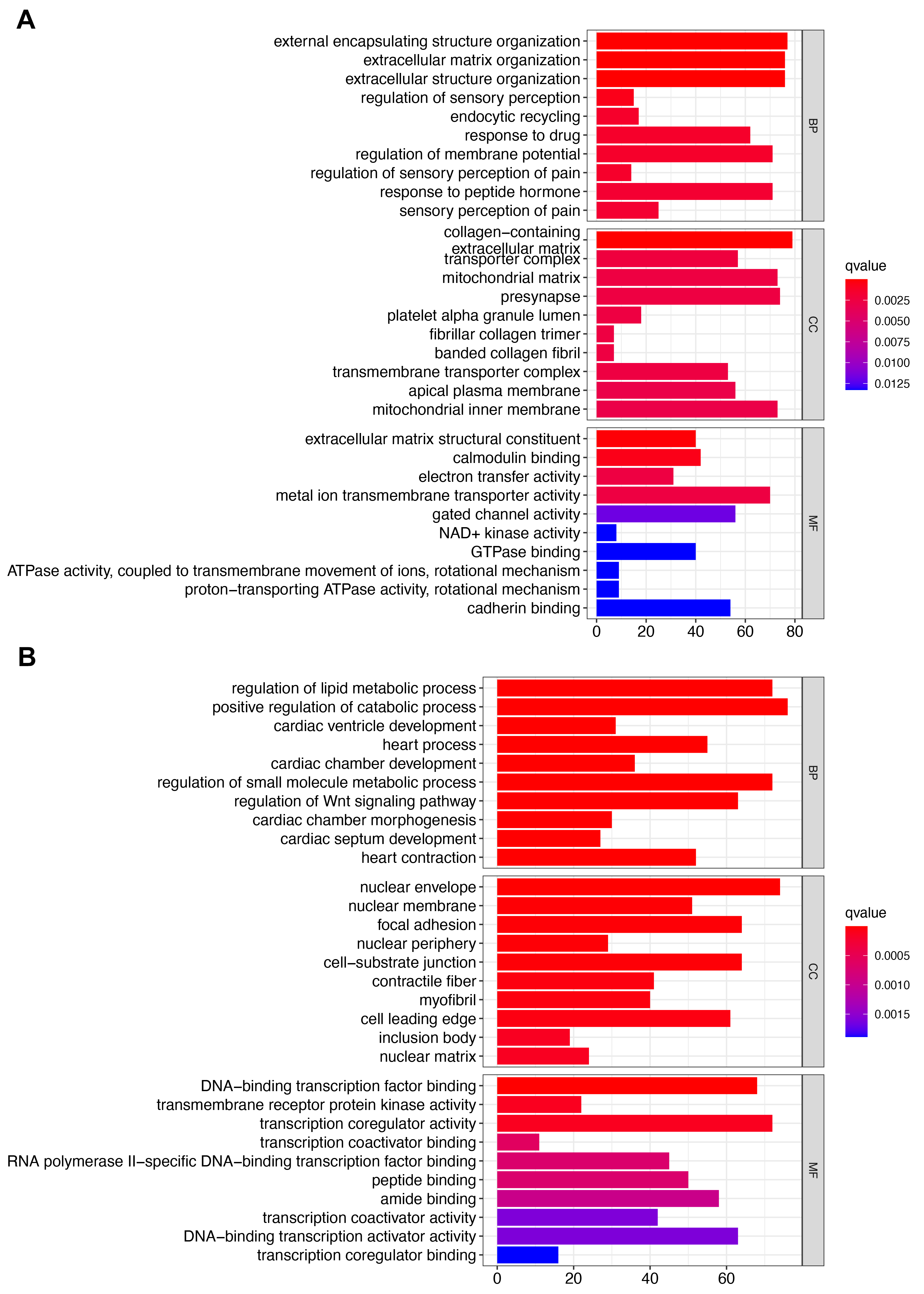


Supplementary Figure 2. Demonstration of GO function analysis of DEmRNAs in AF. (A). GO function up-regulated in the AF group is shown. (B). GO function down-regulated in the AF group is shown.


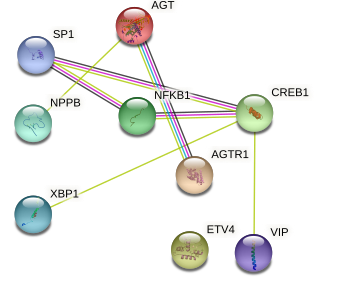


Supplementary Figure 3. Protein-protein interaction network of SP1, AGT, NPPB, NFKB1, CREB1, XBP1, AGTR1, ETV4 and VIP.


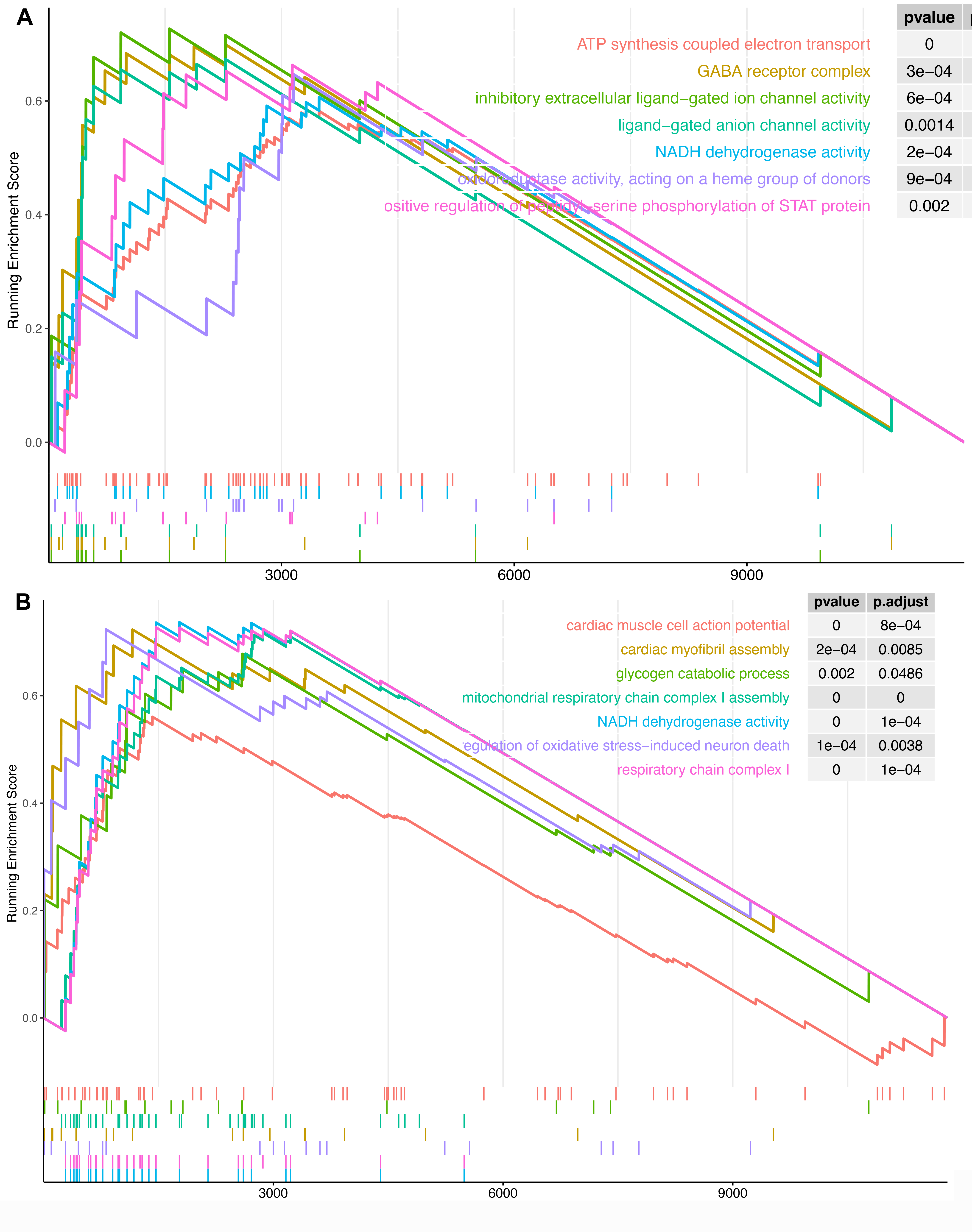


Supplementary Figure 4. GSEA analysis of VIP in AF. (A).Functional enrichment of the CREB1 high expression group in AF. (B).Functional enrichment of VIP high expression group in AF.
